# Do Human Assertions Really Adhere Strictly to Norms? The Effect of Threatening Content in Information on Personalized Norm Perception

**DOI:** 10.3390/bs14070625

**Published:** 2024-07-22

**Authors:** Shijia Zhang, Jiangdong Diao, Jiahui Huang, Yanchi Liu, Lei Mo

**Affiliations:** 1 Center for Studies of Psychological Application, Guangdong Provincial Key Laboratory of Mental Health and Cognitive Science, School of Psychology, South China Normal University, Guangzhou 510631, China; shijia.zhang@m.scnu.edu.cn (S.Z.); 2021023727@m.scnu.edu.cn (J.D.); 2 Shenzhen Pingshan Foreign Languages Wenyuan School, Shenzhen 518000, China; 2019022978@m.scnu.edu.cn; 3 Key Laboratory of Chinese Learning and International Promotion, School of International Culture, South China Normal University, Guangzhou 510631, China

**Keywords:** information dissemination, norm of assertion, threat

## Abstract

Assertion is the use of declarative sentences to convey information, which necessitates meeting the “justified-belief norm” as a prerequisite. However, a significant amount of misinformation that did not meet these conditions was spread during COVID-19, leading to a reintroduction of the assertion norm. One possible hypothesis is that the threatening content of the misinformation influenced the perception of the norm. However, this remains unclear to researchers. Therefore, we conducted two experiments to investigate the effect of threatening content in information on individuals’ perceptions of norms. In all the experiments, participants read backstories with and without threatening content, followed by answering assertion questions. It was observed that people do follow a looser assertion norm for information that contains threatening content. Additionally, further exploration revealed that threatening factors also lead individuals to more easily perceive the related content as truth and reduce the probability of being blamed. These two outcomes provide some explanation for the underlying mechanism of threatening factors’ influence. The research results further refined the theory of assertion norms, offering a certain basis for information management.

## 1. Introduction

### 1.1. The Norm of Assertion and Its Research Progress

Assertion is an important way for humans to use language to share information and it plays a crucial role in society. It means that the speaker conveys information in the form of a declarative or affirmative sentence [1,2]. The norm of assertion refers to the idea that a speaker needs to meet certain conditions before making an assertion [3,4,5,6]. What exactly are the specific conditions required for an assertion? This is an important question in the study of assertion. Previous researchers have extensively explored this topic, identifying four types of norms and comparing them to find the true norm [7]. Before introducing them, it should be noted in advance that these four norms are not philosophical norms. They represent people’s preferences for these norms from a social psychology perspective. This reflects what people tend to follow in personalized norm perception.

Four specifications have been proposed to explain the need for assertions, but one of them has gained more support recently. Early researchers emphasized the importance of knowledge. Knowledge is defined as information that is true and known to the person. Information can only be asserted if it is based on knowledge norms (truth and known: the information must be true and known) [8,9,10,11,12,13,14]. Ref. Wiener (2005) proposes an interesting norm to challenge this notion, emphasizing the truth without regard to whether the parties know it or not (truth: as long as the information is true, it does not matter if the person does not know) [15]. If information is true, it can be asserted even if the parties do not know it. In general, both knowledge and truth norms depend on the truth. These overly strict requirements have been increasingly challenged, especially due to the onslaught of two belief norms (belief: the information may not necessarily be the truth, but the individual must believe the information). One perspective (the belief norm) is that assertions are entirely dependent on beliefs that do not require any justification as a precondition [16,17]. The other norm (the justified-belief norm) (belief and justification: the information may not necessarily be the truth, but the individual must believe the information, and this belief must be justified) emphasizes the necessity for beliefs to be justified as a precondition, such as a vague memory [7,18,19]. Recently, the justified-belief norm has been shown to be more effective than the other three, and the findings are supported by evidence from multiple regions [20,21]. These latest studies resolve the controversy among the four norms, indicating that human assertions are based on justified beliefs rather than on the other three norms.

### 1.2. Justified-Belief Norm Facing Shocks and Potential Hypotheses

According to the results of these studies, assertions (declarative or affirmative sentences) can circulate in society only if they fulfill the preconditions of justification. However, some recent social phenomena have challenged this inference. During the COVID-19 pandemic, there was a surge in false information such as “wearing masks harms your respiratory system” or “vaccines lead to infertility” [22,23]. These assertions are neither true nor justified. How do these assertions break through the barrier of the justified-belief norm to spread across society? It is worth noting that during the COVID-19 pandemic, the disinformation that was widely disseminated in society often had threatening content [24]. Is it possible that threatening content in messages influences assertion norms (personalized norm perception)? Previous researchers were uncertain about this.

Several related studies in other fields have provided evidence for this hypothesis. Numerous studies have observed the different effects of negative information, such as threats, on various aspects of human cognition [25,26]. This hints that the threatening content of information may have different effects on personalized norm perception. More importantly, organisms are often more sensitive to threats [27,28]. This means that organisms are more inclined to accept threatening information. This is consistent with the phenomenon that information containing threatening content is more likely to spread, which provides a physiopsychological-based rationale for this phenomenon. Because of the biological need for security, individuals tend to let threatening information pass through the assertion norm, even without justification. Based on the above, it is reasonable to hypothesize that the norms for assertions corresponding to messages containing threatening content may be more lenient. At the same time, this hypothesis also impacts existing research results [20]. When confronted with information containing threatening content, should we consider justified-belief norms over belief norms?

### 1.3. The Potential Value and Significance of Testing the above Hypotheses

The exploration of the above hypotheses yields positive outcomes in various aspects. Previous research discussions have not considered the impact of threatening content [20,29]. Exploring the effect of threatening content contributes to further supplementing and refining the theory of assertion norms. In addition, the spread of false information has received increasing attention recently [30]. Further verification of the assertion norm is of great significance for a deeper understanding of this important social phenomenon. The results of the psychological and social sciences have an increasing impact on public policy [31], and our exploration can also provide new scientific implications for public policy formulation. Finally, our research may have implications for broader related research topics, such as conspiracy. This is a question that psychology researchers have long been interested in [32]. Conspiracies generally contain threatening content, and their spread may also be related to the lax norm of assertion.

### 1.4. Research Program

Here, we investigate the impact of threatening content on the assertion norm through two behavioral experiments. The basic hypothesis of this study is that, compared with general information without threatening content, information containing threats is more likely to surpass the constraint of personalized norm perception, leading to a more relaxed assertion norm. Both Experiments 1 and 2 are centered on this hypothesis. The distinction lies in the fact that Experiment 1 conducts a fundamental verification based on justified beliefs and belief norms to investigate the role and difference of threatening content under the two norms. Experiment 2 delves deeper into exploring the universality of the impact of threatening content in extreme scenarios and investigates whether the influence of threatening content persists in cases where there are no beliefs.

## 2. Experiment 1

Experiment 1 has two purposes. First, it was designed to examine how the threatening content of the information affects the assertion. To achieve this, we manipulated the argument variable by presenting two types of information (general and threatening). It is important to clarify that threatening information refers to content that contains threats, not information intended to make participants feel threatened. Secondly, it also aims to retest the superiority of the rational belief criterion over the belief criterion in the context of threatening information. Two levels of belief reliability were included for this test (justify belief and belief).

### 2.1. Participants, Materials, and Procedure

The researchers recruited Han or Chinese language-proficient university participants from China through online advertisements (more than 99% were Han Chinese), and the participants received monetary rewards. In line with traditional research methods, the participants engaged in our experiment online through a questionnaire platform without any restrictions or requirements on their experimental equipment or environment [6,20]. At the outset of the experiment, the participants were required to read and sign an informed consent form within the web questionnaire. Subsequently, the participants formally commenced the experiment and proceeded to follow the instructions in the questionnaire to read the material and respond to the questions. A total of 562 individuals passed the attention test, among whom 176 were male, with an average age of 22.58 and a standard deviation of 5.64. The number of participants was consistent with previous studies, which ensures the statistical validity of this study [6]. The experiment was adapted from the classic airport problem [20], a 2 (general, threatening) × 2 (justify belief, belief) between-subjects experiment. The participants were randomly assigned to one of four conditions. The participants were all from China, and the materials they saw during the experiment were all in Chinese. To facilitate dissemination, we followed the approach of previous studies and created an English cultural version [20]. The content of the Chinese and English versions was identical, except for details like names and places. The four conditions were as follows:

- General & Justify Belief—Jack is waiting for a flight to Russia at New York’s Kennedy International Airport. An elderly woman asks him if he can tell her which boarding gate the flight to France departs from. Jack remembers seeing a list of boarding gate information where the only gate listed for flights to France was Gate 24.
- Threatening & Justify Belief—Jack is waiting for a flight to Russia at New York’s Kennedy International Airport. An elderly woman asks him if she can board a flight to France through Gate 24. Jack recalls seeing a notice stating that Gate 24 has serious structural issues and could collapse, potentially harming those passing through.
- General & Belief—Jack is waiting for a flight to Russia at New York’s Kennedy International Airport. An elderly woman asks him if he can tell her which boarding gate the flight to France departs from. Jack couldn’t find any information regarding boarding gates, but he has a feeling that the flight to France leaves from Gate 24, so he believes it might be there.
- Threatening & Belief—Jack is waiting for a flight to Russia at New York’s Kennedy International Airport. An elderly woman asks him if she can board a flight to France through Gate 24. Jack didn’t see any notice, but suddenly has a premonition that there might be a risk of the building collapsing as the woman passes through Gate 24, so he believes she might be in danger.

The participants were shown one of the stories and then asked to answer a series of questions, including two assertion questions, one fact question, one belief question, and one question regarding reasonableness.

In the general condition, the questions were as follows:

- Assertion Question 1—Do you think Jack can say, “The flight to France is at Gate 24”? (Yes/No)
- Assertion Question 2—Do you think Jack should say, “The flight to France is at Gate 24”? (Yes/No)
- Truth Question—Is the boarding gate for the flight to France really at Gate 24? (Yes/No)
- Belief Question—Does Jack believe that the boarding gate for the flight to France is at Gate 24? (Yes/No)
- Justify Question—Does Jack’s belief that “the flight to France is at Gate 24” have reasonable evidence? (Yes/No)

In the threatening condition, the questions were as follows:

- Assertion Question 1—Do you think Jack can say, “There’s danger at Gate 24”? (Yes/No)
- Assertion Question 2—Do you think Jack should say, “There’s danger at Gate 24”? (Yes/No)
- Truth Question—Is there really a possibility of danger at Gate 24? (Yes/No)
- Belief Question—Does Jack believe that the elderly woman might encounter danger passing through Gate 24? (Yes/No)
- Justify Question—Does Jack’s belief that “the elderly woman might encounter danger” have good evidence? (Yes/No)

### 2.2. Results

**Figure 1:**
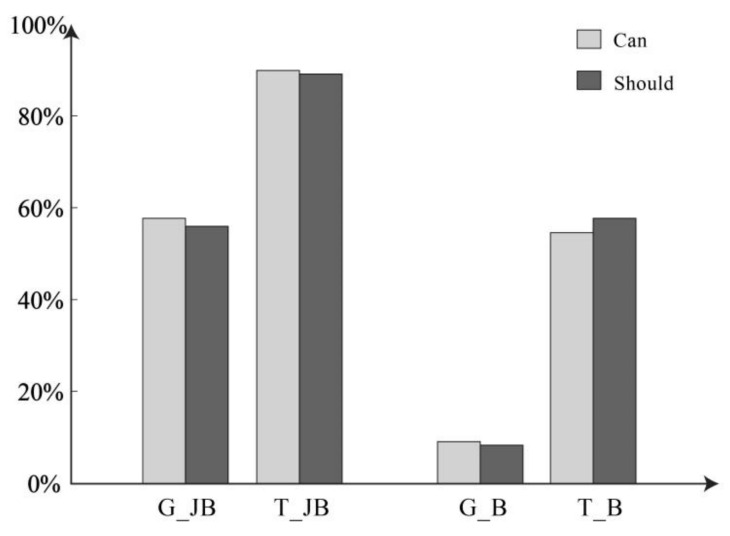
Judgments of assertability across formulations and scenarios. In the figure is the probability of choosing “Yes”. G_JB is General & Justify Belief; T_JB is Threatening & Justify Belief; G_B is General & Belief (no Justify); T_B is Threatening & Belief (no Justify).

Assertion Question 1—Do you think Jack can say…? In the condition where there is reasonable evidence, 57.80% of individuals in the general (non-threatening) group chose “Yes”. In the threatening group, 90.00% of individuals chose “Yes”. In the condition without reasonable evidence, 9.16% of individuals in the general group chose “Yes”, and 54.69% of individuals in the threatening group chose “Yes”. The statistical graphic is shown in Figure 1. Further analysis showed that the differences in belief reliability were statistically significant (*χ*^2^ = 89.544, *p* < 0.001), as were the differences in information type (*χ*^2^ = 71.194, *p* < 0.001). The results from the logistic regression indicated a significant main effect of belief reliability. In the condition of reliable belief, the likelihood of selecting “Yes” increased by 7.457 times compared to the unreliable belief condition (*B* = 2.009, *p* < 0.001, *OR* = 7.457 [95% *CI:* 3.814–14.579]). Additionally, there was a significant main effect of information type. In the threatening condition, the likelihood of selecting “Yes” increased by 6.570 times compared to the general condition (*B* = 1.883, *p* < 0.001, *OR* = 6.570 [95% *CI:* 3.438–12.555]). The interaction effect was not significant (*B* = 0.600, *p* = 0.213).

Assertion Question 2—Do you think Jack should say…? Under the condition of having a reasonable basis, 56.07% of individuals in the general group chose “Yes”. In the threatening group, 89.23% of individuals chose “Yes”. Under the condition of lacking a reasonable basis, 8.40% of individuals in the non-threat group chose “Yes”, while 57.81% of individuals in the threat group chose “Yes”. The statistical graphic is shown in Figure 1. Further analysis showed that the differences in belief reliability were statistically significant (*χ*^2^ = 78.745, *p* < 0.001), as were the differences in information type (*χ*^2^ = 81.405, *p* < 0.001). The results from the logistic regression indicated a significant main effect of belief reliability. In the condition of reliable belief, the likelihood of selecting “Yes” increased by 6.046 times compared to the unreliable belief condition (*B* = 1.799, *p* < 0.001, *OR* = 6.046 [*95% CI:* 3.137–11.654]). Additionally, there was a significant main effect of information type. In the threatening condition, the likelihood of selecting “Yes” increased by 6.492 times compared to the general condition (*B* = 1.871, *p* < 0.001, *OR* = 6.492 [95% *CI:* 3.455–12.197]). The interaction effect was not significant (*B* = 0.834, *p* = 0.085).

**Figure 2:**
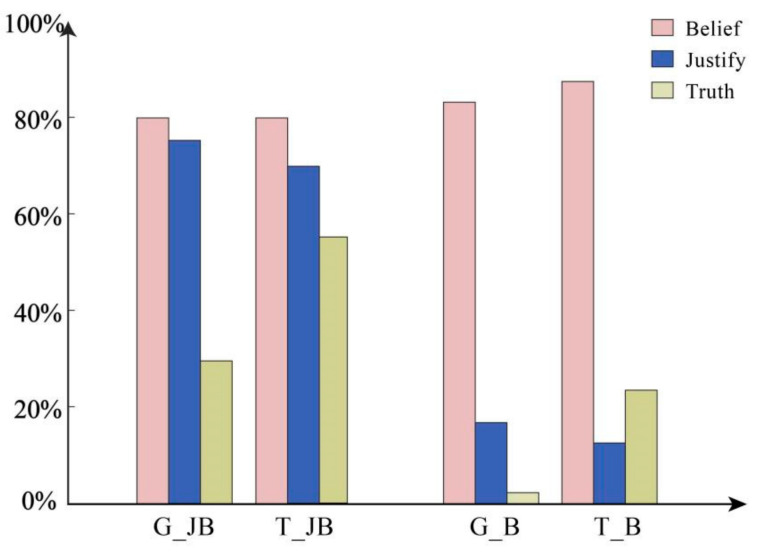
Belief (Yes), Justify (Yes), and Truth (Yes) questions.

Besides the primary outcomes, the researchers also conducted additional validation on participants’ comprehension and reading proficiency, as depicted in Figure 2.

Firstly, based on the text design in this article, regardless of whether there is a reasonable basis or not, the character “Jack” believes in the statements he makes. In other words, “Jack” himself believes in his assertions. The experiment examined this through belief-related questions. Binomial distribution testing revealed that across all four conditions, participants’ probability of choosing “Yes” was significantly higher than the chance probability of 50%, *p* < 0.001. The results indicate that participants’ choices were not random.

Secondly, this study examined participants’ judgments regarding the rationality of beliefs under different conditions. The results revealed that under the condition of having a belief, participants’ probability of choosing “Yes” was significantly higher than the chance probability of 50%, *p* < 0.001. Conversely, under the condition of lacking a belief, participants’ probability of choosing “Yes” was significantly lower than the chance probability of 50%, *p* < 0.001. The logistic regression results indicated that in reliable belief conditions, compared to unreliable belief conditions, participants’ probability of choosing “Yes” increased by 15.163 times (*B* = 2.719, *p* < 0.001, *OR* = 15.163 [95% *CI:* 9.900–23.223]). These findings demonstrate the successful manipulation of different conditions in our study.

The two aforementioned results indicate the scientific validity of the entire study. Additionally, the researchers explored an intriguing question: to what extent do participants believe that an assertion is factual and whether it is affected by the presence of threatening content in the information. The results from the chi-square test revealed statistically significant differences in belief reliability (*χ*^2^ = 52.958, *p* < 0.001) and information type (*χ*^2^ = 31.951, *p* < 0.001). The logistic regression results demonstrated a significant main effect of belief reliability, where the probability of choosing “Yes” increased by 3.931 times in reliable belief conditions compared to unreliable belief conditions (*B* = 1.369, *p* < 0.001, *OR* = 3.931 [95% *CI*: 2.302–6.714]). There was also a significant main effect of information type, where the probability of choosing “Yes” increased by 2.879 times in threat conditions compared to non-threat conditions (*B* = 1.057, *p* < 0.001, *OR* = 2.879 [95% *CI*: 1.789–4.631]). The interaction effect was significant (*B* = 1.512, *p* < 0.05, *OR* = 4.537 [95% *CI*: 1.230–16.737]). A simple effect analysis revealed a significant difference between threat and non-threat conditions in unreliable belief conditions, where the threat condition was 13.061 times more likely than the non-threat condition (*B* = 2.570, *p* < 0.001, *OR* = 13.061 [95% *CI*: 3.873–44.048]). In reliable belief conditions, the difference was also significant, but the threat condition was only 2.879 times more likely than the non-threat condition (*B* = 1.057, *p* < 0.001, *OR* = 2.879 [95% *CI:* 1.789–4.631]). The interaction observed in Figure 2 mainly stems from the very low probability of choosing “Yes” in the condition of non-threat and unreliable belief.

### 2.3. Discussion

Based on the results of Experiment 1, the conclusions from previous studies (purpose 1) and the main hypotheses of this research have all been validated (purpose 2).

Specifically, there is a reasonable belief that indeed serves as a key factor influencing assertions; information with justified beliefs does indeed tend to generate assertions more easily, consistent with prior research [7]. Experiment 1 observed that, even in information containing threatening content, the presence of justification remained a key factor. This study addresses the impact of disinformation dissemination on the norm of justified beliefs and responds to this important question. In addition, our study was conducted in China to validate the belief criterion, which complements the work of Kneer’s study (2021), which was tested in the United States, Germany, and Japan [20].

More importantly, the results of Experiment 1 validated our hypothesized key effect by observing the effect of threatening content in information on individuals’ perceived norms. Threatening factors similarly exerted an influence; compared to general information, information that contains threatening content more readily elicits assertions. These main findings hold true across both classic questioning approaches (“can” and “should”). This has important implications for explaining and responding to the spread of disinformation.

It is worth noting that Experiment 1 also revealed a tendency for individuals to equate justified beliefs with facts, supporting the notion of “justified beliefs as a basis for assertions” replacing the idea of “facts as a basis for assertions” [20]. Additionally, in this context, threatening information also played a role, guiding individuals to perceive it as more factual.

## 3. Experiment 2

Experiment 1 observed that threatening information enhances assertiveness, irrespective of whether the participants’ beliefs are rational. Experiment 2, building upon Experiment 1, further investigates whether the threatening content of the information can influence individuals when they lack beliefs. Different information types (general and threatening) and varying degrees of belief (believing and hesitating) were established.

### 3.1. Participants, Materials, and Procedure

Researchers recruited Han or Chinese language-proficient participants through online platforms (more than 99% were Han Chinese), and the participants received monetary rewards. Participants were no longer limited to universities but came from a broader sample of society. A total of 405 individuals passed the attention test (including 172 males, with an average age of 30.84 and a standard deviation of 7.70). This was a 2 (general, threatening) × 2 (believing, hesitating) between-subjects experiment where participants were randomly assigned to one of four conditions. Other details are the same as in Experiment 1. All four conditions lacked reasonable justification:

- General Condition & Belief—Mary stands at the entrance of an ophthalmology hospital. A young person asks her if this hospital performs the LINU surgery to improve eyesight. Mary responds, “This hospital doesn’t perform the LINU surgery”. It’s important to note that Mary isn’t certain if the hospital does perform this surgery. She heard one claim stating the hospital lacks doctors skilled in this technique, but later heard another claim that the hospital does possess this technology. Nevertheless, Mary firmly believes the hospital doesn’t possess the LINU technology.
- Threatening Condition & Belief—Mary stands at the entrance of an ophthalmology hospital. A young person asks her if the LINU technique for improving eyesight is safe. Mary responds, “The LINU technique isn’t safe”. It’s important to note that Mary isn’t sure about the safety of the LINU technique. She heard one claim stating the LINU technique isn’t safe and might lead to deteriorating eyesight after a dozen years. However, she later heard another claim stating LINU is safe. Overall, Mary firmly believes LINU is an unsafe technique.
- General Condition & No Belief (hesitation)—Mary stands at the entrance of an ophthalmology hospital. A young person asks her if this hospital performs the LINU surgery to improve eyesight. Mary responds, “This hospital doesn’t perform the LINU surgery”. It’s important to note that Mary isn’t certain if the hospital performs this surgery. She heard one claim stating the hospital lacks doctors skilled in this technique, but later heard another claim that the hospital does possess this technology. In conclusion, Mary feels uncertain about the hospital’s possession of the LINU technology, her belief vacillates.
- Threatening Condition & No Belief (hesitation)—Mary stands at the entrance of an ophthalmology hospital. A young person asks her if the LINU technique for improving eyesight is safe. Mary responds, “The LINU technique isn’t safe”. It’s important to note that Mary isn’t sure about the safety of the LINU technique. She heard one claim stating the LINU technique isn’t safe and might lead to deteriorating eyesight after a dozen years. However, she later heard another claim stating LINU is safe. In conclusion, Mary feels uncertain about the safety of the LINU technique, her belief vacillates.

The participants were presented with one of the stories and then asked to respond to a series of questions, including two assertion questions, one factual question, one belief question, and one blame question.

Additionally, Experiment 1 largely employed binary yes-or-no forced-choice tasks for both the factual and belief questions. In real life, individuals often exhibit uncertainty in their understanding of facts and beliefs. For instance, they might not be certain whether something is a fact or not. Therefore, for these two problems in Experiment 2, we provided Yes, Uncertain, and No options. At the same time, it was found in Experiment 1 that both the “should” and “can” paradigms that assert problem 1 achieved the same result. Therefore, only one of the paradigms was kept in Experiment 2, and a new questioning paradigm, the “permit” paradigm, was introduced. Being permitted is a looser requirement than “should” and “could” [7].

In the general condition, the questions were as follows:

- Assertion Question 1: Should Mary say “This hospital cannot perform the LINU surgery”? (Yes/No)
- Assertion Question 2: Is Mary permitted to say “This hospital cannot perform the LINU surgery”? (Yes/No)
- Truth Question: Can this hospital really not perform the LINU surgery? (Yes/Uncertain/No)
- Belief Question: Does Mary believe “This hospital cannot perform the LINU surgery”? (Yes/Uncertain/No)
- Blame Question: Do you think Mary’s behavior should be blamed? (Yes/No)

In the threatening condition, the questions were as follows:

- Assertion Question 1: Should Mary say “LINU surgery is unsafe”? (Yes/No)
- Assertion Question 2: Is Mary permitted to say “LINU surgery is unsafe”? (Yes/No)
- Truth Question: Is LINU surgery really unsafe? (Yes/Uncertain/No)
- Belief Question: Does Mary believe “LINU surgery is unsafe”? (Yes/Uncertain/No)
- Blame Question: Do you think Mary’s behavior should be blamed? (Yes/No)

### 3.2. Results

**Figure 3:**
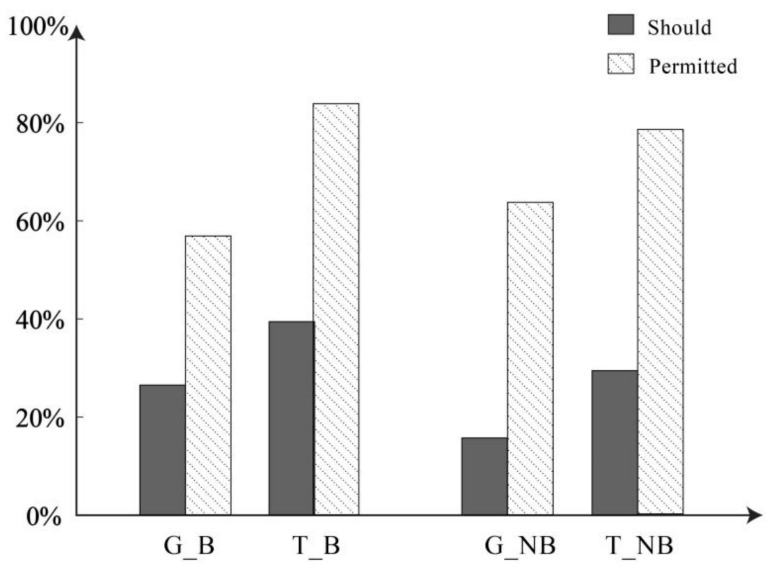
Judgments of assertability across formulations and scenarios. In the figure is the probability of choosing “Yes”. G_B is General Condition & Belief; T_B is Threatening Condition & Belief; G_NB is General Condition & No Belief (hesitation); T_NB is Threatening Condition & No Belief (hesitation).

Assertion Question 1—Should Mary say…? The observation made in our study is that, in the assertion question, when there is belief, 26.47% of the participants in the no-threat group chose “Yes”. In the threat group, 39.39% chose the same response. When there is no belief, 15.69% of the no-threat group and 29.41% of the threat group chose “Yes”. The statistical graph is shown in Figure 3. The difference between belief and disbelief is statistically significant (*χ*^2^ = 5.355, *p* < 0.05), and there is also statistical significance in the difference between information types (*χ*^2^ = 8.884, *p* < 0.01). Further logistic regression analysis reveals that the main effect of belief is not significant (*B* = −0.445, *p* = 0.137). However, the main effect of information type is significant, indicating that the likelihood of saying “Yes” increases by 2.240 times in the threat condition compared to the no-threat condition (*B* = 0.806, *p* < 0.05, *OR* = 2.240 [95% *CI*: 1.131–4.433]). The interaction effect is not significant (*B* = −0.215, *p* = 0.085).

Assertion Question 2—Is Mary permitted to say…? Under the condition of belief, 56.86% of individuals in the no-threat group chose to permit such a statement, whereas 83.84% in the threat group chose to permit it. In the absence of belief, 63.73% in the no-threat group and 78.43% in the threat group chose to permit such a statement. The statistical graph is shown in Figure 3. Further analysis indicated that the difference in permitting such a statement between belief and disbelief did not have statistical significance (*χ*^2^ = 0.042, *p* = 0.837), while the difference in information type was statistically significant (*χ*^2^ = 21.111, *p* < 0.001). Consequently, the logistic regression model, excluding belief and focusing solely on information type as the independent variable, revealed a significant main effect for information type. Specifically, in the presence of threatening content in the information compared to no-threat conditions, the likelihood of permitting such a statement increased by a factor of 2.825 (*B* = 1.038, *p* < 0.001, *OR* = 2.825 [95% *CI:* 1.800–4.434]).

**Figure 4:**
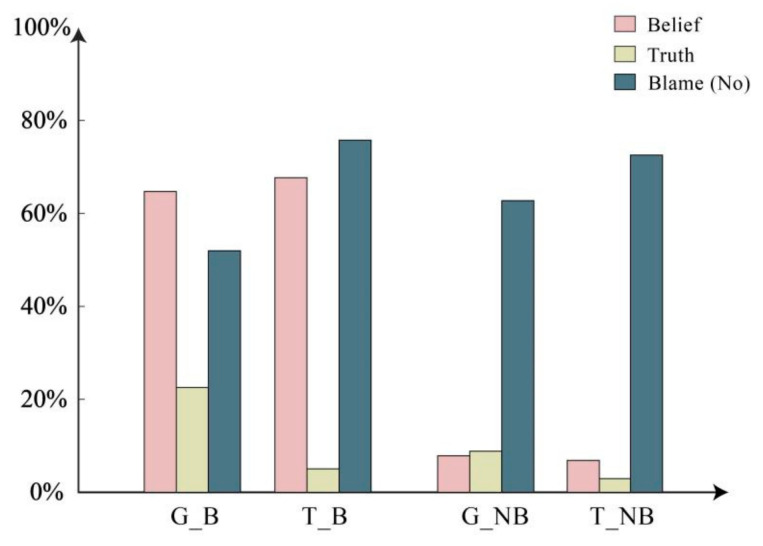
Belief (Yes), Truth (Yes), and Blame (No) questions.

**Figure 5:**
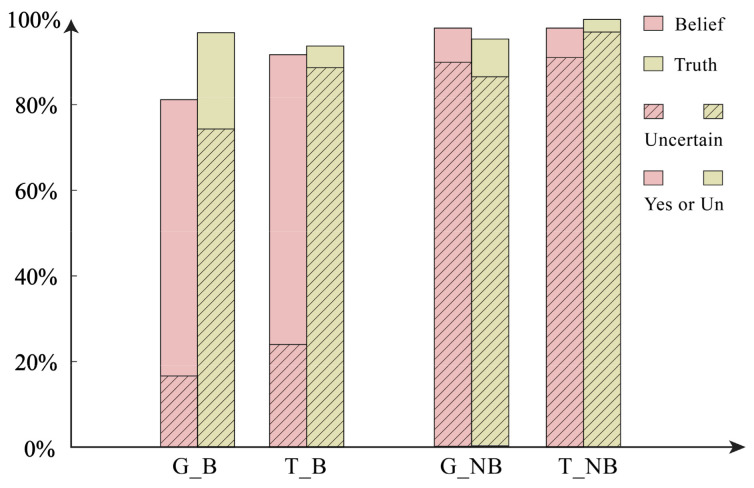
Different answers (Yes or Uncertain) to Belief and Truth across scenarios. The stripes in the figure are the probabilities of choosing “Uncertain” in both the factual question and the belief question. A solid color block represents the sum of the probabilities of choosing “Yes” and “Uncertain”.

Beyond the primary results, the researchers further validated participants’ comprehension and reading levels, as illustrated in Figure 4 and Figure 5.

Firstly, based on the text design of this study, the character “Mary” is not clear about the truth. Therefore, in the factual questions, Mary is uncertain about the facts. There are three options in the factual questions (Yes, Uncertain, and No), with Yes and No combined into one option. A binomial distribution test was performed on the processed data (the probability of choosing Yes or No is 66.666%, while the probability of choosing Uncertain is 33.333%). The results indicate that in all four scenarios, the probability of participants choosing non-factual information is significantly higher than the chance level of 33.333%, *p* < 0.001. The participants’ choices were not random.

This study observed that the participants who read the text attentively and made deliberate choices were able to distinguish between having belief and not having belief. The results indicated that in the question of whether the person had belief, the group with belief significantly chose “Yes” more often than the chance probability of 33.333%, *p* < 0.001. Conversely, the group without belief significantly chose “Yes” less often than the chance probability of 33.333%, *p* < 0.001. A logistic regression revealed that the absence of belief significantly increased the likelihood of choosing “Uncertain” relative to having belief. The probability of choosing “Uncertain” was 40.008 times higher when not having belief compared to having belief (*B* = 3.689, *p* < 0.001, *OR* = 40.008 [95% *CI*: 22.289–71.813]). These findings indicate successful manipulation of different conditions in our study.

The two aforementioned results demonstrate that the overall design of our study was scientific. Additionally, the researchers delved into an intriguing question: whether the assertion of blame is a more morally charged issue compared to statements like “should say”, “can say”, or “permitted to say”. Some actions may not be permitted but might not necessarily lead to the individual being blamed. The researchers further explored whether such a broader question could also be influenced by threat. The findings revealed that the difference between having and not having belief did not hold statistical significance (*χ*^2^= 1.530, *p* = 0.216). However, there was statistical significance in the difference in information types (*χ*^2^= 19.656, *p* < 0.001). Based on the chi-square test results, in the logistic regression, belief was not considered, and only information type was included as the independent variable. The results indicated a significant main effect of information type; in the threat condition compared to the non-threat condition, the probability of choosing “No” increased by 2.063 times (*B* = 0.724, *p* < 0.001, *OR* = 2.063 [95% *CI*: 1.494–2.848]).

### 3.3. Discussion

The results of Experiment 2 once again validated the efficacy of threatening factors. In the absence of justified beliefs throughout, the primary effect of threat remains consistently significant. Compared to general information, threatening information more readily prompts assertions. Such outcomes hold true across both paradigms (“should” and “permitted”). Additionally, the no-belief condition implies an assertion that does not rely on any preconditions, which is extremely lenient. In such a condition, the threatening content of the message still has an effect on the individual’s perception of the assertion, which further demonstrates the stability of the effect of threatening content.

Of interest is the fact that differences in paradigms affect individuals’ perceptions of assertion norms. In the “should” paradigm, assertions are judged below chance probability due to a lack of justification. However, in the more relaxed permissive paradigm, the permissibility of assertions behind them does not depend on justification as a prerequisite. This difference, while not negating the importance of justification for the assertion, creates a shock. This suggests that researchers should focus on the role of different paradigms in various story contexts.

Additionally, an intriguing exploration was discovered. Information under threatening conditions, if it triggers assertions, is less likely to be reproached compared to information devoid of threatening factors. This further validates a crucial hypothesis of this paper, as mentioned in the introduction: Even without reliable reasons, individuals tend to heed threatening information and allow or assist in its dissemination.

## 4. General Discussion

### 4.1. The Effect of Threatening Content on Individuals’ Perceptions of Assertions

Two different contexts and multiple assertion paradigms were utilized to examine the influence of threatening content in information on assertion norms. The primary finding is that, when faced with information containing threatening content, more participants chose to assert that it should, can, or is permitted to be stated. This effect was consistent across the various contexts and paradigms. These results suggest that threatening content in information causes individuals to relax their personal perception of norms. Our study resolves the contradiction between prior research on assertion norms and real-world scenarios. The dissemination of misinformation, such as the “vaccine threat theory”, which lacks empirical evidence, is exacerbated by the inclusion of threatening content [24].

Moreover, further exploration revealed that threatening content not only prompts assertions more readily but also enhances the perceived truthfulness of related information (Experiment 1). Additionally, it mitigates reproach from individuals (Experiment 2). These findings provide insights into the underlying mechanisms through which threatening content influences assertion norms. These novel findings and the further exploration of mechanisms are of significant value, contributing to a deeper understanding of the cognitive mechanisms underlying personal assertion perceptions. Individuals tend to apply more lenient assertion standards to such misinformation, perceiving them as true more readily and showing less condemnation. This study effectively addresses gaps in assertion norm theory research, indicating that while assertions follow the “justified-belief norm”, they are also influenced by additional contextual factors.

### 4.2. The Effect of Threatening Content on “Justified-Belief Norm”

As mentioned in the introduction, if the “justified-belief norm” consistently outperforms the other three norms, particularly the “belief norm”, then messages lacking justified beliefs would violate assertion norms and not be widely disseminated. However, reality contradicts this. Does this mean that threatening content in false messages undermines the “justified-belief norm” and supports the “belief norm” instead? We found that, although threatening content does have an impact, beliefs with justified reasons are still more assertable than those lacking justified reasons (Experiment 1; Experiment 2 “should”). The theory of the “justified-belief norm” remains robust and aligns with previous research findings [20,21].

However, the results from the permissive paradigm yield different answers. In this paradigm, assertions are allowed even in the absence of reliable beliefs. This difference may be related not only to the questioning paradigm but also to the context of the story containing the medical theme in Experiment 2. This suggests that researchers need to further explore how the permissive paradigm functions in different narrative contexts. Permission itself is a relaxation of the individual’s perceived norms, as is the role of threatening content. The combination of the two may have contributed to this result. In general, our findings serve as a critical supplement to recent important research, carrying significant theoretical implications.

### 4.3. Potential Inspiration for Other Research Questions Arising

In addition, our research not only contributes to academic studies on assertion norms but also has various other social and theoretical implications. Managing the spread of misinformation is a critical public issue [22]. Our study further explains the reasons for the spread of disinformation, providing scientific justification and insights for implementing additional constraints on disinformation. In addition, our study observed that assertion relaxation was associated with less blame, which also provides insight into constraining disinformation. Awakening a sense of responsibility in the population seems to be a good approach.

Additionally, this study contributes valuable insights into certain psychological phenomena. The prevalence and beliefization of conspiracy theories have long fascinated researchers in psychology [32]. It is important to explore the cause and process. Our study provides important insights into how the threatening content of conspiracy theories may contribute to their spread and persistence and warrants further research. In general, the current research provides important insights for addressing both social issues and research questions.

### 4.4. Research Gaps and Possible Research Directions

Finally, this study bears certain limitations as it solely observes the impact of threatening content in the information on assertion norms. In contrast, it is unclear whether the assertion of the asserted judge’s own threat perception affects the individual’s norm perception. In the context of the COVID-19 pandemic, some individuals experienced anxiety or depression [33]. This negative emotional feeling may be a factor in their adoption of a more lenient assertion norm. In previous assertion studies [20], the assertion judge has typically adopted a third-party perspective without utilizing various textual materials to evoke diverse emotional responses in the participants themselves. Consequently, the exploration of this aspect remains inconclusive. Future research could delve into this crucial issue comprehensively.

## Data Availability

The data used in the study can be made available upon requests addressed to the corresponding author.
